# Microperipheral Iridectomy for Troublesome Posterior Synechiolysis in Secondary Intraocular Lens Implantation

**DOI:** 10.1155/2021/6634871

**Published:** 2021-02-23

**Authors:** Wu Xiang, Jing Li, Wan Chen, Haotian Lin, Weirong Chen

**Affiliations:** State Key Laboratory of Ophthalmology, Zhongshan Ophthalmic Center, Sun Yat-sen University, Guangzhou 510060, China

## Abstract

**Purpose:**

To introduce an effective method for separating extensive posterior synechiae and those located under or adjacent to surgical incisions.

**Methods:**

Pediatric patients who had been subjected to cataract surgery and developed troublesome posterior synechiae requiring secondary intraocular lens (IOL) implantation were recruited. All patients underwent microperipheral iridectomy at the 12 o'clock position. Then, an ophthalmic viscosurgical device was injected into the posterior chamber through the iris fistula to mechanically separate the posterior synechiae, using scissors to cut robust posterior synechiae if necessary. The results of posterior synechiolysis and the position of the implanted IOL were analyzed.

**Results:**

Sixteen patients (median age, 51.56 months; range, 28–80 months) were included. The scope of posterior synechia in clock was 4.42 (range, 1–10). All troublesome posterior synechiae were successfully separated using the microperipheral iridectomy method, and all patients underwent IOL implantation in the ciliary sulcus. There was one case of peripheral iridectomy-related early intraoperative bleeding; no bleeding was observed at the end of surgery.

**Conclusions:**

Microperipheral iridectomy is a useful method for the management of troublesome posterior synechiae during secondary IOL implantation in pediatric patients, which makes secondary IOL implantation an easier and safer method in some challenging cases.

## 1. Introduction

Pediatric cataract is the leading cause of blindness in children, and timely cataract surgery is crucial for restoring vision. Following defects in the blood-aqueous barrier, the pediatric eye may develop severe postoperative inflammatory reactions, which may result in multiple complications, such as visual axis opacification [1, 2], severe inflammatory response [3, 4], formation of secondary membranes [3], intraocular lens (IOL) precipitates [5], pupillary capture [5], posterior synechiae [5], corectopia [6], glaucoma [1, 7], endophthalmitis [6], and retinal detachment [8, 9], among other conditions. Due to the inherent nature of the developing eyes and the narrow anterior ocular segment in children, not all pediatric patients can immediately receive IOL implantation after cataract surgery, particularly those aged <2 years [10]. Therefore, some pediatric patients may suffer from aphakia following cataract surgery. Although optical correction in patients with aphakia can be managed with contact lenses or spectacles, IOL implantation is often indicated in older patients in order to improve vision [11]. Therefore, in older patients, secondary IOL implantation is often required.

To ensure appropriate IOL positioning, one of the most important factors is to dilate the pupil as much as possible, until a capsular condition is observed. However, the presence of posterior synechiae may restrict pupil dilation. Therefore, it is necessary to separate the posterior synechiae in order to allow the enlargement of the pupil prior to IOL implantation. Insufficient posterior synechiolysis may lead to dislocated IOL, eccentrically positioned IOL, pupillary capture, and deformed pupil, which may cause symptoms such as the edge effect, marked reduction in visual acuity, diplopia, glare [12], decreased focal depth, and the release of potential inflammatory mediators [13], thus requiring additional surgery. Local posterior synechiae and synechiae that are located at a site distant from the primary incision are usually easily manageable. However, it is more challenging to separate extensive posterior synechiae and those that are located under or adjacent to the surgical incision, which are defined as troublesome posterior synechiae (TPS) in the present study.

Peripheral iridectomy is a classic procedure in glaucoma surgery, which allows the communication between the anterior and posterior chambers, thus expanding the anterior chamber and reducing the incidence of anterior chamber angle closure. Interestingly, peripheral iridectomy was first performed in 1948 by Hilding to prevent iris prolapse during cataract surgery [14]. We found that peripheral iridectomy not only creates a communication between the posterior and anterior chambers but may also serve as a passage to separate TPS, facilitating optimal IOL implantation and ensuring central placement of the IOL. The present study was performed with the aim to introduce microperipheral iridectomy as a useful method for posterior synechiolysis, particularly for TPS in pediatric patients who require secondary IOL implantation.

## 2. Methods

### 2.1. Study Participants

Between March 2015 and December 2018, we retrospectively collected 16 patients (19 eyes) who required secondary IOL implantation and presented with TPS after previous pediatric cataract surgery. The baseline data of the patients, including age, sex, surgical eye, results of posterior synechiolysis, and position of the implanted IOL were analyzed. Complications, visual acuity, and intraocular pressure were recorded at the last follow-up after surgery. All surgeries were recorded. The study was performed in accordance with the principles of the Declaration of Helsinki and with the approval of the Ethics Committee of Zhongshan Ophthalmic Center, Guangzhou, China. Informed consent was obtained from each participant.

### 2.2. Microperipheral Iridectomy and Posterior Synechiolysis

A scleral tunnel incision was made above the 12 o'clock position. An ophthalmic viscosurgical device (OVD) was injected into the anterior chamber to protect the corneal endothelium and maintain the structure of the anterior chamber. A forceps was used to pull the peripheral iris out of the anterior chamber, and a small peripheral iridectomy was made under the surgical incision using scissors. The OVD was injected into the posterior chamber through the iris fistula, and the posterior synechiae were mechanically separated. Scissors were used to cut the posterior synechiae if necessary. According to the condition of the residue capsule, the IOL was implanted in the ciliary sulcus or capsular bag, and the fibrosis of the iris or capsule was removed with a vitrector if necessary. The detailed demonstration of posterior synechiolysis by microperipheral iridectomy is shown in Supplementary Video 1.

## 3. Results

**Figure 1:**
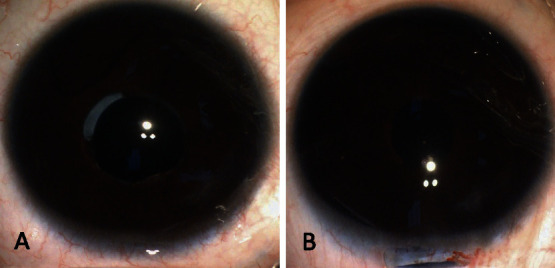
Representative anterior segment images before and after posterior synechiolysis. (a) Preoperative image of extensive and superior posterior synechia. Position, 6–3 o'clock. (b) Postoperative condition of (a), showing a round pupil, a centrally placed IOL, and a barely noticeable peripheral iridectomy.

**Figure 2:**
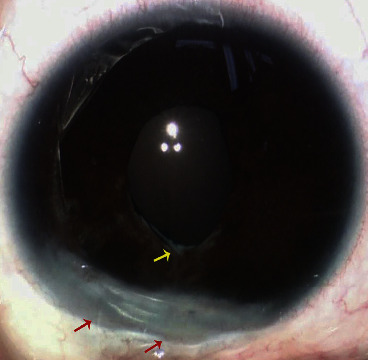
The anterior segment of the patient who exhibited intraoperative bleeding. This case presented with superior corneal leukoma (red arrows), superior posterior synechiae with fibrous membrane (yellow arrow), and peripheral anterior synechiae.

Sixteen pediatric patients (19 eyes) who required secondary IOL implantation and presented with TPS were recruited in the present study. The baseline characteristics of the patients are summarized in Table 1. The mean age of the patients was 52.89 ± 17.05 months (range, 28–80 months). The mean scope of the posterior synechiae in clock positions was 4.42 ± 2.61 (range, 1–10). All TPS were successfully separated, and IOL was implanted in the ciliary sulcus or capsular bag without intraoperative or postoperative complications. Representative anterior segment images before and after posterior synechiolysis are presented in Figure 1. One patient developed peripheral iridectomy-associated early intraoperative bleeding that spontaneously stopped after ∼1 min. The anterior segment of this patient exhibited peripheral anterior synechia and corneal leukoma (Figure 2). No intraocular hypertension was observed in the collected patients at the last follow-up visit.

## 4. Discussion

Pediatric cataract surgery is usually accompanied by an inflammatory reaction that may cause TPS. In addition, damaging or irritating the iris during surgery may greatly increase postoperative inflammation, which markedly increases the risks of a secondary IOL implantation. The presence of posterior synechiae may restrict pupil dilation, making it more difficult to observe the intraocular conditions and evaluate the residue capsules which will support the implanted IOL. It is crucial to understand whether there are sufficient capsules to support the implanted IOL, and implanting an IOL in the ciliary sulcus or capsular bag without knowing the state of the capsules may have a severe negative impact on the patient, as the IOL may reach the vitreous cavity, intra or postoperatively. In addition, it was previously reported that existing iridocapsular adhesions may compromise IOL implantation in the sulcus [11]. Therefore, it is imperative to separate the posterior synechiae in order to achieve a round pupil, allowing adequate visualization of the intraocular conditions before selecting the optimal position of the IOL (which may be implanted in the sulcus or fixed to the sclera or to the anterior chamber) to avoid severe complications.

In the literature, there are several reported methods that may be used to perform synechiolysis. Focal posterior synechia may be treated by initially performing a dissection using a 27-gauge needle [15], spatula [13], or Nd:YAG laser [13]. By contrast, extensive and sticky iridolenticular adhesions may be separated using vitreoretinal scissors through two corneal paracenteses, whereas fibrosis of the iris and capsule may be removed with a vitrector [15]. However, the use of the vitrector is limited due to its high cost.

Peripheral iridectomy was first introduced by Albrecht Von Graefe in 1857 [16], and it has been a routine procedure in traditional glaucoma surgery to facilitate the flow of the posterior aqueous fluid into the anterior chamber [17]. In addition, Hilding hypothesized that iris prolapse in cataract surgery may be caused by the high pressure of the posterior chamber and successfully addressed this problem by performing a peripheral iridectomy, which was carried out by making an incision, thus reducing the pressure and allowing the release of the aqueous fluid [14]. In the present study, we introduced a method for separating TPS by performing peripheral iridectomy. As expected, all TPS were easily separated via peripheral iridectomy, and this method may also reduce the damage of intraocular tissue due to excessive manipulation following inadequate pupil dilation.

In our procedure, peripheral iridectomy was defined as microperipheral iridectomy, as the aim of this method was to create an iris fistula as small as possible. This method allowed the reduction of the size of the iris fistula in order to insert only the required surgical instruments, reducing the iris damage, in contrast with the traditional glaucomatous iridectomy. Our results demonstrated that TPS can be easily separated using this method. In addition, using this method made it possible to assess the condition of the residual capsule in order to decide whether to implant the IOL in the ciliary sulcus. All patients in the present study exhibited a successfully implanted IOL in the ciliary sulcus or capsular bag using this method, and no intraoperative or postoperative complications were observed, which may be attributed to this microperipheral iridectomy method allowing complete separation of the posterior synechia and careful examination of the residue capsule. In previous studies, the optimal IOL position was reported to be the capsular bag [18], particularly for pediatric eyes [19]. However, almost all IOLs were implanted in the ciliary sulcus rather than the capsular bag in our study, which was due to the sticky fused anterior-posterior capsulotomy edges and the large defects in the center posterior capsule. To reduce the incidence of visual disturbances, the position of the peripheral iridectomy is usually located in the superior part of the eye in patients with glaucoma. In this study, the preferential site of the IOL was above the 12 o'clock position, as the iris fistula could be covered by the upper eyelid. In addition, the incision size of the peripheral iridectomy was as small as possible, and it was barely noticeable after surgery. In addition, Srinivasan et al. [20] reported that peripheral iridectomy is likely safe with respect to visual dysphotopsias, regardless of the location and size. Therefore, separating the posterior synechiae using the microperipheral iridectomy method barely causes any visual abnormalities. One of the patients developed bleeding during peripheral iridectomy, which stopped spontaneously after ∼1 min. The pathophysiological characteristics of this patient were analyzed, and we found that this patient presented a peripheral anterior synechia and corneal leukoma, which may be combined with abnormal vessels and cause hemorrhage during mechanical separation. Whether this complication was associated with the younger age of the patient requires further investigation. In addition, prednisolone acetate ophthalmic suspension and tropicamide eye drops were used after surgery in our study, which greatly reduced posterior synechia recurrence and severe postoperative inflammation. As some patients cannot corporate with vision examination, we did not describe visual acuity information.

This was a retrospective study; the main limitation was the limited number of subjects examined. In order to validate whether this method should be used routinely in pediatric patients, a prospective study with a larger sample size is required.

## 5. Conclusions

In summary, microperipheral iridectomy is a useful strategy for the management of TPS during secondary IOL implantation in pediatric patients. Although all cases in the present study were pediatric patients, we consider that this method may also be applied for other conditions and populations presenting with TPS. In addition, although peripheral iridectomy is helpful for managing posterior synechiae, it would be more meaningful to avoid irritating the iris and focus on postoperative anti-inflammatory treatment following pediatric cataract surgery.

## Figures and Tables

**Table 1:** Demographic and clinical details of the patients.

Variables	Value
No. of patients (no. of eyes)	16 (19)

Age at secondary IOL implantation (months)	
Mean	51.56 ± 16.08
Range	28–80

Scope of posterior synechiae (clock hours)	
Mean	4.42 ± 2.61
Range	1–10

Position of IOL (no. of eyes)	
Ciliary sulcus	18
Capsular bag	1

Follow-up period (months)	
Mean	14.90 ± 14.59
Range	1–38

Posterior synechiae recurrence	0

IOL, intraocular lens.

## Data Availability

The datasets analyzed during the current study are available from the corresponding author upon request.

## Abbreviations

OVD: Ophthalmic viscosurgical device
IOL: Intraocular lens implantation
TPS: Troublesome posterior synechiae.

## Supplementary Materials

Supplementary Materials The detailed demonstration of posterior synechiolysis by microperipheral iridectomy is shown in Supplementary Video 1.
