# Supplementary material for: Microperipheral Iridectomy for Troublesome Posterior Synechiolysis in Secondary Intraocular Lens Implantation
Source: J Ophthalmol. 2021 Feb 23;2021:6634871. doi: 10.1155/2021/6634871 (PMC7929652; doi:10.1155/2021/6634871)
Supplement: Supplementary Materials — The detailed demonstration of posterior synechiolysis by microperipheral iridectomy is shown in Supplementary Video 1. [file 6634871.f1.docx]

Supplementary video 1:

https://drive.google.com/file/d/1G9BEwPxn39VV-LXkEr92R_uYcqA89zSv/view?usp=sharing
